# Anti-CD70 Immunocytokines for Exploitation of Interferon-γ-Induced RIP1-Dependent Necrosis in Renal Cell Carcinoma

**DOI:** 10.1371/journal.pone.0061446

**Published:** 2013-04-17

**Authors:** Peirong Chen, Shoko Nogusa, Roshan J. Thapa, Calvin Shaller, Heidi Simmons, Suraj Peri, Gregory P. Adams, Siddharth Balachandran

**Affiliations:** 1 Immune Cell Development and Host Defense Program, Fox Chase Cancer Center, Philadelphia, Pennsylvania, United States of America; 2 Developmental Therapeutics Program, Fox Chase Cancer Center, Philadelphia, Pennsylvania, United States of America; 3 Department of Biostatistics and Bioinformatics, Fox Chase Cancer Center, Philadelphia, Pennsylvania, United States of America; Johns Hopkins School of Medicine, United States of America

## Abstract

Metastatic renal cell carcinoma (RCC) is an incurable disease in clear need of new therapeutic interventions. In early-phase clinical trials, the cytokine IFN-γ showed promise as a biotherapeutic for advanced RCC, but subsequent trials were less promising. These trials, however, focused on the indirect immunomodulatory properties of IFN-γ, and its direct anti-tumor effects, including its ability to kill tumor cells, remains mostly unexploited. We have previously shown that IFN-γ induces RIP1 kinase-dependent necrosis in cells lacking NF-κB survival signaling. RCC cells display basally-elevated NF-κB activity, and inhibiting NF-κB in these cells, for example by using the small-molecule proteasome blocker bortezomib, sensitizes them to RIP1-dependent necrotic death following exposure to IFN-γ. While these observations suggest that IFN-γ-mediated direct tumoricidal activity will have therapeutic benefit in RCC, they cannot be effectively exploited unless IFN-γ is targeted to tumor cells *in vivo*. Here, we describe the generation and characterization of two novel ‘immunocytokine’ chimeric proteins, in which either human or murine IFN-γ is fused to an antibody targeting the putative metastatic RCC biomarker CD70. These immunocytokines display high levels of species-specific IFN-γ activity and selective binding to CD70 on human RCC cells. Importantly, the IFN-γ immunocytokines function as well as native IFN-γ in inducing RIP1-dependent necrosis in RCC cells, when deployed in the presence of bortezomib. These results provide a foundation for the *in vivo* exploitation of IFN-γ-driven tumoricidal activity in RCC.

## Introduction

Renal cell carcinomas (RCC) account for approximately 3% of all adult cancers [1]. Several histological subtypes of RCC have been described; of these the Clear Cell variant (ccRCC) represents the primary subtype, and accounts for up to 85% of all RCC cases [2], [3]. For most patients with early-stage RCC, surgery as monotherapy or as part of a multimodal treatment plan remains the standard of care and offers excellent five-year survival rates [4]. Unfortunately, RCC is largely asymptomatic, and about a third of all patients have locally-advanced or metastatic disease at presentation. Unlike localized early-stage disease, metastatic RCC is an invariably fatal cancer and the most lethal of all genitourinary neoplasms [1], [5].

Current frontline treatment options for metastatic RCC center around small-molecule inhibitors of cell-growth, angiogenesis, and nutrient-sensing pathways, but these agents only delay disease progression and are not curative [6], [7], [8]. Before the introduction of pharmacological approaches, cytokine-based immunotherapy – IFN-α and IL-2 in particular – represented the primary treatment options for RCC [9], [10], [11]. Approximately 5–20% of patients with metastatic RCC show partial responses to immunotherapy, with complete responses reported in a smaller subset. Indeed, the curative ability of cytokine-based approaches remains the primary advantage of immunotherapy over chemotherapy, despite the severe side effects that often accompanies use of these biological agents in the clinic [9], [10], [11].

To a large extent, the ability of cytokines to provide lasting remission may stem from their ability to activate multiple anti-tumor mechanisms. For example, the cytokine IFN-γ is not only immunomodulatory, but also anti-angiogenic and, relevant to this study, directly tumoricidal [12], [13]. Our laboratory is interested in exploiting IFN-γ as an anti-RCC therapeutic by focusing on its direct tumoricidal properties. We have identified the transcription factor NF-κB as a survival mechanism that, when disabled, renders otherwise-resistant mammalian cells susceptible to RIP1-kinase-dependent necrotic death following direct exposure to IFN-γ [14].

Constitutively elevated NF-κB activity appears to be a common occurrence in ccRCC [15], [16], and disabling NF-κB signaling in these cells, for example, by using the proteasome inhibitor bortezomib, sensitizes them to multiple anti-neoplastic agents, including apoptosis by the cytokine TRAIL and oncolysis by the RNA virus encephalomyocarditis virus [17], [18], [19], [20], [21]. Bortezomib is thought to function as an NF-κB inhibitor at least in part by preventing proteasomal degradation of the NF-κB inhibitory protein I-κB [22], [23].

Taking advantage of the observations that (1) NF-κB protects cells from IFN-γ, (2) NF-κB is a survival factor in RCC, and (3) one mechanism by which bortezomib mediates its anti-tumor effects is by inhibiting NF-κB, we have found in preliminary experiments that bortezomib renders a panel of RCC cell lines susceptible to IFN-γ-induced necrosis at doses of each agent that are physiologically very achievable (RJT, PC, and SB, unpublished data). While these pre-clinical observations strongly suggest that the combination of IFN-γ and bortezomib (or other NF-κB inhibitors) will have therapeutic benefit in ccRCC, they cannot be successfully exploited unless IFN-γ has direct access to RCC cells *in vivo*. Given that IFN-γ has a very short half-life in serum, and manifests significant toxic side effects following systemic administration, achieving bioactive concentrations of IFN-γ at the tumor represents a significant clinical challenge [24], [25].

A potential solution to the twin issues of IFN-γ’s poor stability and systemic toxicity can be found in the recently developed class of biotherapeutics called ‘immunocytokines’, or antibody-cytokine conjugates [26]. IFN-γ-based immunocytokines can not only target IFN-γ to RCC cells, but can also improve its *in vivo* stability by taking advantage of the prolonged half-life of intact antibodies in circulation, a property conferred on immunoglobulins via interaction between their Fc domains and the salvage receptor FcRn [27]. Typically, immunocytokines are created by fusing the cytokine to the carboxyl-terminus of an antibody heavy chain, sterically distant from the antigen-binding site and thus unlikely to interfere with tumor targeting. The cytokine is attached to the antibody heavy chain by a polypeptide linker that is not only flexible enough to allow engagement of the cytokine with its receptor, but is also resistant to serum proteases that might otherwise prematurely unlink the cytokine from the antibody. As antibodies contain two heavy chains, fusing cytokines to the heavy chain results in an agent with two cytokine moieties per antibody [26].

Epstein and colleagues have generated IFN-γ immunocytokines by fusing IFN-γ to a tumor-targeting antibody (TNT-3) [28], [29]. Encouragingly, they report that such IFN-γ immunocytokines not only improve IFN-γ’s half-life, but also greatly increase its bioavailability at solid tumors [28], [29]. TNT-3, however, recognizes DNA released from dying cells, and as such does not target IFN-γ to living tumor cells [30]. In this study, we therefore sought to generate immunocytokines that will selectively target IFN-γ to live RCC cells such that its tumoricidal properties can be exploited.

Here, we report the development and characterization of immunocytokines in which either human or murine IFN-γ is fused to an antibody targeting the putative metastatic ccRCC biomarker CD70 [31]. CD70 is the membrane-bound ligand of the Tumor Necrosis Factor Receptor (TNFR) superfamily member CD27 [32]. While CD70 is found on over 60% of ccRCC samples tested, its expression in metastatic ccRCC samples approaches a remarkable 100% [33]. Importantly, CD70 is mostly undetectable in normal kidney tissue and highly-restricted in its expression in other normal tissues, making it an ideal antigen for immunocytokine targeting strategies in RCC [31], [33], [34], [35].

We show that anti-CD70-targeted IFN-γ immunocytokines display robust IFN-γ activity, bind several human RCC cell lines, and kill RCC cells by RIP1-dependent necrosis, when combined with bortezomib. These findings represent key steps in the development of second-generation IFN-γ-based therapeutic approaches for RCC.

## Materials and Methods

### Cell Lines and Reagents

RCC cell lines ACHN, Caki-1, 786-0, 769-P and RenCa were obtained from the ATCC, cultured in EMEM (ACHN), McCoy’s 5a (Caki-1), or RPMI-1640 (786-O, 769-P and RenCa) media supplemented with 10% FBS and antibiotics, and used within six months of resuscitation. The ccRCC-derived HRC63 cell line was established at the Fox Chase Cancer Center as described previously [36], and cultured in IIA medium (DMEM/F12 supplemented with 1.2 g/ml NaHCO_3_, 1.6 μM FeSO_4_, 50 nM sodium selenite, 25 μg/ml insulin, 200 nM hydrocortisone, 10 μg/ml transferrin, 1 nM triiodothyronine, 10 μU/ml vasopressin, 10 nM cholesterol, 10 ng/ml epidermal growth factor, and 15% FBS). Cytokines and chemicals were from the following sources: human and murine IFN-γ (R&D Systems), Necrostatin-1 (gift of A. Degterev, Tufts University), Bortezomib (Millennium). Primary antibodies were purchased from Upstate (pSTAT1), Santa Cruz (anti-Myc), BD Biosciences (STAT1), Sigma (β-Actin), Ancell (anti-CD70-FITC), and Roche (Rituximab, obtained through the Fox Chase Cancer Center Pharmacy). FITC-labeled secondary anti-human IgG antbodies for FACS were purchased from Life Technologies.

### Cloning of Immunocytokine cDNA Expression Vectors

**Figure 1:**
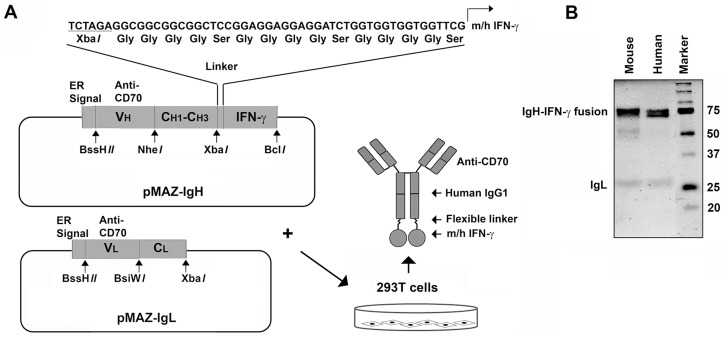
Generation and purification of mIFN-γ and hIFN-γ immunocytokines targeting CD70. (A) Two plasmids – pMAZ-IgH and pMAZ-IgL – were used as backbones to construct and express anti-CD70 immunocytokines bearing either murine (m) or human (h) IFN-γ. pMAZ-IgH expresses the anti-CD70 heavy chain separated from murine or human IFN-γ by a flexible (Gly)_4_-Ser linker. pMAZ-IgL encodes the anti-CD70 light chain. For details of construction, expression and purification, please see the Materials and Methods section. (B) Coomassie Blue-stained SDS-PAGE gel of mIFN-γ-anti-CD70 immunocytokine (lane 1), and hIFN-γ-anti-CD70 immunocytokine (lane 2) purified from supernatants of 293T cells after transfection with the plasmids described in A.

DNA expression cassettes corresponding to the coding sequences of either human or murine IFN-γ, each with an in-frame (Gly)_4_-Ser linker were synthesized by BioGen and cloned into the XbaI-BclI site of the plasmid pMAZ-IgH [37] to generate pMAZ-IgH-hIFN-γ or pMAZ-IgH-mIFN-γ. The heavy chain complementarity determining region (CDR) from an anti-human CD70 monoclonal antibody developed for therapy by Seattle Genetics (clone 1F6, [38]) was similarly synthesized by BioGen and cloned into the BssHII-NheI sites of pMAZ-IgH-hIFN-γ and pMAZ-IgH-mIFN-γ. The resultant vectors were designated pMAZ-IgH-anti-hCD70-hIFN-γ or pMAZ-IgH-anti-hCD70-mIFN-γ. Finally, the light chain CDR from the same anti-human CD70 monoclonal antibody was synthesized by BioGen and cloned into the BssHII-BsiWI site of pMAZ-IgL to generate pMAZ-IgL-anti-hCD70 (see Fig. 1A for schematic; pMAZ vectors are a kind gift of I. Benhar, Tel Aviv University, Israel).

### Generation of Immunocytokines in 293T Cells

To produce immunocytokines, HEK 293T cells (1×10^7^) in T525 flasks were transfected with 60 μg of pMAZ-IgL-anti-hCD70 together with either 60 μg of pMAZ-IgH-anti-hCD70-hIFN-γ or pMAZ-IgH-anti-hCD70-mIFN-γ by using 300 μg of polyethylenimine (1 mg/mL, pH7.4; polyethylenimine, linear; M.W. 25000, Polysciences, Inc.). Supernatants from these cells were collected every 24–36 h for 6–7 days post-transfection, clarified by centrifugation (1400×g, 10 minutes at 4°C) then subjected to protein A affinity chromatography (Pierce Protein A Plus Agarose). Immunocytokines were eluted in IgG elution buffer (pH2.8, Pierce) and immediately neutralized with Tris (1M, pH9.0), dialyzed against PBS, and stored in aliquots at −80°C.

### Immunoblot Analyses

Whole-cell extracts from cells (8×10^5^/well) plated in six-well plates were prepared in TL buffer (1% [v/v] Triton-X100, 150 mM NaCl, 20 mM HEPES [pH 7.3], 5 mM EDTA, 5 mM NaF, 0.2 mM NaVO_3_ [*ortho*], and Complete Protease Inhibitor cocktail [Roche]) and clarified by centrifugation (15000×g, 10 minutes at 4°C). Samples were denatured by boiling in Laemmli buffer (0.1% [v/v] 2-mercaptoethanol, 0.0005% [w/v] bromophenol blue, 10% [v/v] glycerol, 2% [w/v] SDS, 63 mM Tris-HCl [pH 6.8]) for 2 minutes and separated by 12% SDS-PAGE. Protein gels were transferred onto PVDF membranes (Millipore) and blocked in blocking buffer (0.1% [v/v] Tween20, 5% [w/v] non-fat dry milk in PBS) at room temperature for 15 minutes. Blots were incubated for at least 18 h at 4°C with primary antibody (p-STAT1, 1∶500; STAT1, 1∶2000; Myc, 1∶2000) diluted in blocking buffer, followed by 3 washes for 10 minutes each in washing buffer (0.1% [v/v] Tween 20 in PBS) at room temperature. Next, blots were incubated with secondary antibody (horseradish peroxidase-conjugated goat anti-rabbit/mouse IgG (H+L), 1∶5000, Jackson ImmunoResearch) for at least 18 h at 4°C, followed by 3 washes of 10 minutes each in washing buffer at room temperature. Blots were then incubated in enhanced chemiluminescence substrate (Pierce ECL Plus Western Blotting Substrate, Pierce) for one minute, and proteins were detected by chemiluminescence using X-ray film (Denville Scientific Inc.).

### IFN-γ Antiviral Assay

Caki-1 or RenCa cells (5×10^5^/well) were plated in six-well plates and allowed to adhere for 24 h before treatment with immunocytokines or recombinant IFN-γ for a further 16 h. Cells were then infected with VSV-GFP [39] in serum-free medium for 45 minutes, following which protection against virus replication was determined microscopically by inhibition of cytopathic effect and GFP fluorescence 20 h post-infection. Virus titers from these cells were quantified by standard plaque assay on immortalized murine embryo fibroblasts, as described previously [40].

### Flow Cytometric Detection of CD70

2×10^5^ cells (HEK 293T, 786-O, 769-P, Caki-1, or ACHN) were harvested from logarithmically-growing cultures with Hanks Buffered Saline Solution/EDTA, washed once with FACS buffer (1% [w/v] BSA in PBS, 0.02% [w/v] NaN3) at 4°C, centrifuged for 5 minutes at 150×g, and re-suspended in 100 μL FACS buffer. Cells were then incubated with 5 μg primary antibody (i.e. human or murine IFN-γ immunocytokines, isotype control human IgG1 [Rituximab], or FITC-conjugated anti-CD70 antibody) for 30 minutes on ice. The cells were then washed twice with FACS buffer and, as necessary, incubated with FITC-labeled secondary anti-human IgG antibody. Samples were examined by flow cytometry on a FACscan flow cytometer (Becton & Dickson), and data analyzed by FloJo 8.8.6 software (Tree Star).

### Statistics

Bar graphs were generated using GraphPad Prism 4 software. Experiments were repeated at least three times, with similar results. For the data showing mRNA expression levels in patient samples, p-values were obtained from moderated t-statistics implemented in LIMMA [41].

## Results

### Generation of Anti-CD70-IFN-γ Immunocytokines

To produce fusion antibodies for the express purpose of targeting IFN-γ to live RCC cells, we developed a two-step cloning strategy using the pMAZ human antibody-producing vectors as backbone (depicted schematically in Fig. 1A). First, we cloned an expression cassette encoding either human or mouse IFN-γ (both with an N-terminal flexible Gly-Ser linker) in frame with the C-terminus of a human IgG heavy chain constant region encoded by pMAZH vector. Next, we used these plasmids to insert heavy-chain CDRs targeting the putative metastatic RCC biomarker CD70. After generating an IgG light-chain expression vector encoding CDRs to CD70, we co-transfected the engineered mIFN-γ or hIFN-γ fusion pMAZH vectors together with pMAZL in 293T cells to generate IFN-γ immunocytokines, each with specificity for human CD70, but bearing C-terminal fusions of either mIFN-γ or hIFN-γ. The IFN-γ immunocytokines were purified from the supernatant of transfected 293T cells by single-step protein A-agarose affinity chromatography and analyzed by denaturing SDS-polyacrylamide gel electrophoresis (SDS-PAGE). Both human and murine IFN-γ-IgG heavy chain fusion polypeptides displayed sizes of ∼70 kDa, consistent with the additional ∼ 15–20 kDa mass of glycosylated IFN-γ over unmodified (∼55 kDa) IgG heavy chain (Fig. 1B). As expected, both light chains migrated equivalently, at ∼25 kDa (Fig. 1B).

Murine and human IFN-γ orthologs share only ∼40% sequence similarity, and do not signal through each other’s receptors (i.e. their activity is species-specific) [42], so our rationale for generating both hIFN-γ- and mIFN-γ-expressing fusions was that each fusion antibody will serve as a control for the other in future *in vivo* experiments. For example, if hIFN-γ- but not mIFN-γ-immunocytokines synergized with bortezomib to inhibit growth of human RCC xenografts in immune-competent mice, we can conclude that direct cytotoxicity by hIFN-γ on RCC cells, rather that indirect immunomodulation by mIFN-γ on murine immune cells, was responsible for therapeutic effect.

### Anti-CD70-IFN-γ Immunocytokines Induce Species-specific Phosphorylation of STAT1

**Figure 2:**
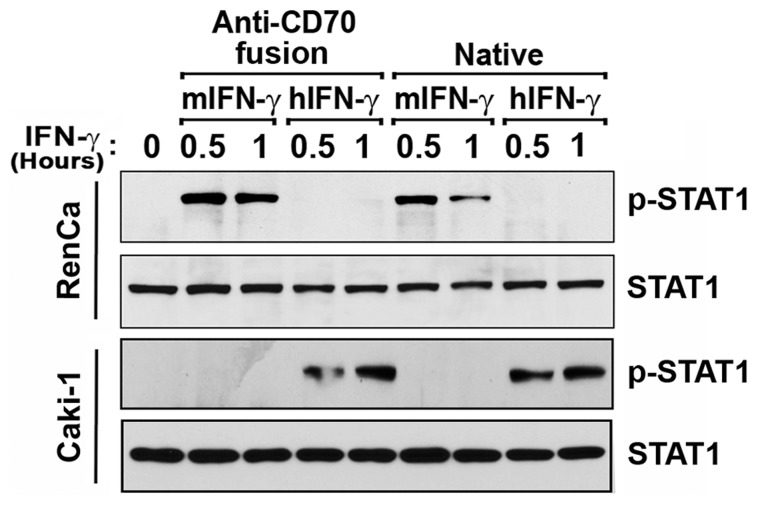
Anti-CD70-IFN-γ immunocytokines induce species-specific phosphorylation of STAT1. Murine (RenCa) or human (Caki-1) RCC cells were treated with anti-CD70 immunocytokines bearing either murine (m) or human (h) IFN-γ (‘Anti-CD70 fusion’, 50 ng/ml). As controls, parallel populations of these cells were treated with recombinant murine or human IFN-γ (‘Native’, 10 ng/ml). At the indicated times post-treatment, cells were examined by immunoblotting for either phosphorylated (p-STAT1) or total STAT1.

Having confirmed expression of each immunocytokine, we next sought to establish their functionality. Focusing first on the cytokine moiety of these fusions, we asked if IFN-γ fused to anti-CD70 antibodies retained activity comparable to unfused, recombinant IFN-γ. To this end, we added treated either human Caki-1 or murine RenCa RCC cells with IFN-γ immunocytokines and measured STAT1 phosphorylation on tyrosine (Y) 701 as evidence of IFN-γ activity [42], [43]. As controls, we incubated these cells with equimolar amounts of either anti-CD70 antibody alone, recombinant human IFN-γ, or recombinant murine IFN-γ. Given that each immunocytokine bears two IFN-γ moieties, and estimating that antibodies are approximately ten times as massive as IFN-γ (∼200 kDa vs. ∼20 kDa), we reasoned that a 1∶5 w/w ratio of native cytokine to immunocytokine will represent molar equivalency (i.e. 1 ng/ml IFN-γ ≈ 5 ng/ml immunocytokine). As shown in Fig. 2, each immunocytokine induced robust, species-specific phosphorylation of STAT1 within 30 minutes of treatment, comparable to what is seen with recombinant IFN-γ preparations. Anti-CD70 antibodies not fused to IFN-γ, or IFN-γ immunocytokines added to species-mismatched cells, did not induce any detectable STAT1-phosphorylation, demonstrating that Y701 phosphorylation was specific to IFN-γ, and confirming the potential usefulness of each fusion as a control for the other in future experiments.

### Anti-CD70-IFN-γ Immunocytokines Display Species-specific Antiviral Activity

**Figure 3:**
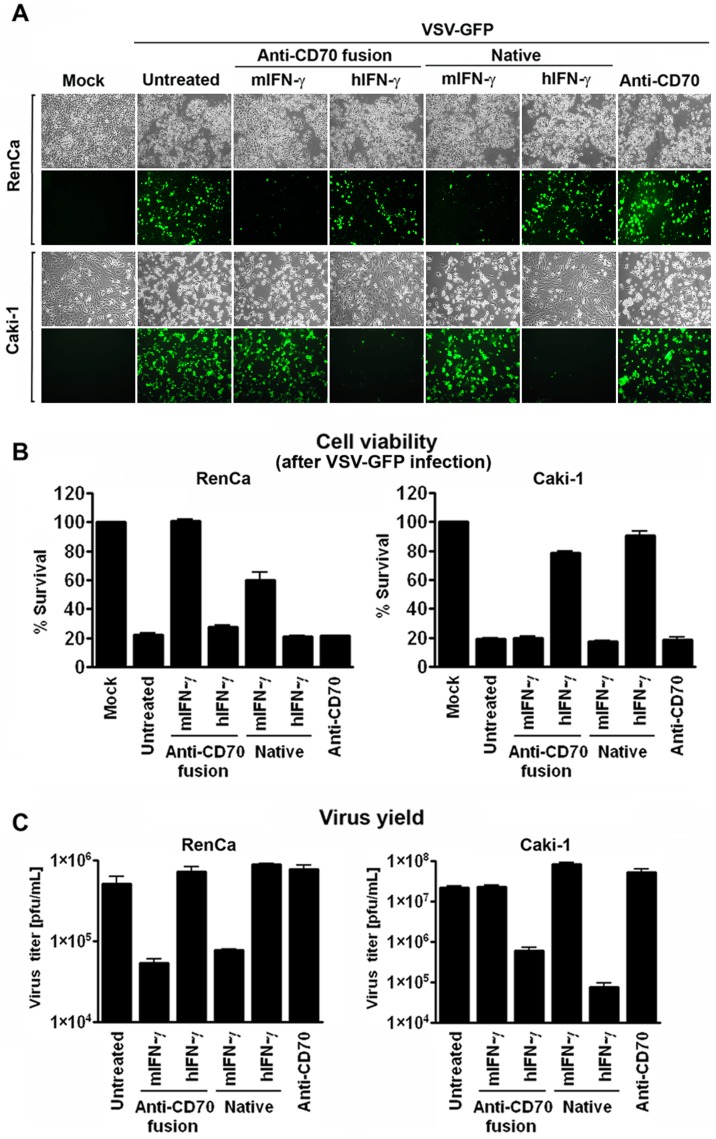
Anti-CD70-IFN-γ immunocytokines display species-specific antiviral activity. Murine (RenCa) or human (Caki-1) RCC cells were pre-treated for 16 h with anti-CD70 immunocytokines bearing either murine (m) or human (h) IFN-γ (‘Anti-CD70 fusion’, 50 ng/ml). As controls, parallel populations of these cells were pre-treated for 16 h with recombinant murine or human IFN-γ (‘Native’, 10 ng/ml), or with unfused anti-CD70 antibody (50 ng/ml). Following pre-treatment, cells were infected with VSV-GFP (MOI = 5 for RenCa, 0.05 for Caki-1). (A) Infected cells were photographed by brightfield (for demonstration of cytopathic effect) or by fluorescence (to show viral replication) microscopy 20 h post-infection. (B) Viability of cells treated as above was determined 20 h post-infection. (C) VSV progeny yield from supernatants of infected cells was determined by standard plaque assay 20 h post-infection. Error bars represent mean +/− S.D, n = 3.

IFN-γ is a well-described antiviral cytokine, capable of inducing a potent STAT1-dependent non-permissive state in target cells. To test if phosphorylation of STAT1 by IFN-γ immunocytokines translated into functional antiviral activity, we pre-incubated Caki-1 and RenCa cells with IFN-γ immunocytokines and, as controls, with equimolar concentrations of unfused anti-CD70 antibody, recombinant murine IFN-γ, or recombinant human IFN-γ. After overnight incubation, by which time the IFN-induced antiviral state is typically well-established, we challenged these cells with vesicular stomatitis virus expressing GFP (VSV-GFP). VSV, a member of the *Rhabdoviridae* family of RNA viruses, is well-known for its sensitivity to IFN [44], [45], and its replication is potently inhibited by IFN-γ pre-treatment [46], [47]. When three indicators of VSV replication – GFP fluorescence, cell death, and progeny virion yield - were examined 20 h post-infection, each was found to be significantly and selectively reduced in cells treated with their cognate species-specific IFN-γ immunocytokine. In particular, the immunocytokines almost completely ablated GFP fluorescence in VSV-GFP-infected cells (Fig. 3A), increased the survival of these cells from ∼20% to over 80% (Fig. 3B), and reduced virus progeny output at least ten-fold (Fig. 3C). The extent to which each immunocytokine inhibited VSV replication was comparable to that achieved by native IFN-γ, demonstrating that IFN-γ activity was not compromised by the process of fusion to an antibody heavy chain.

### Anti-CD70-IFN-γ Immunocytokines Bind Human CD70

**Figure 4:**
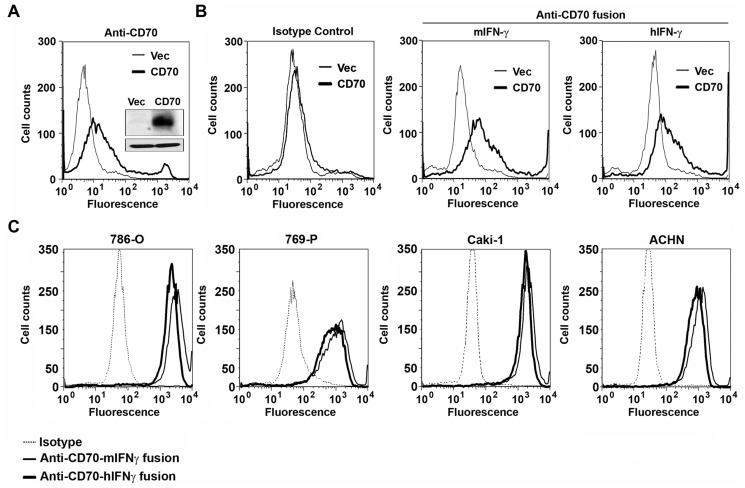
Anti-CD70-IFN-γ immunocytokines bind human CD70. (A) 293T cells were transfected with an expression vector encoding Myc-tagged human CD70 (‘CD70’), or with an empty vector (‘Vec’). 24 h post-transfection, cells were examined for CD70 expression in lysates by anti-Myc immunoblotting (inset, top panel; β-actin loading control, bottom panel), or on the cell surface by FACS staining with a FITC-conjugated anti-CD70 monoclonal antibody. (B) 293T cells were transfected as in A with either an empty vector (‘Vec’) or an expression vector encoding Myc-tagged CD70 (‘CD70’). 24 h post-transfection, cells were incubated with either Rituximab as an isotype control human IgG1 antibody (‘Isotype Control’, left panel), or with immunocytokines bearing either murine (m) or human (h) IFN-γ (‘Anti-CD70 fusion’), and, following labeling with FITC-conjugated anti-human IgG secondary antibodies, analyzed by FACS for CD70 expression. (C) The ATCC-derived RCC cell lines 786-O, 769-P, Caki-1, and ACHN were incubated with either an isotype control human IgG1 antibody (Rituximab, dashed line), anti-CD70-mIFN-γ immunocytokine (thin solid line), or anti-CD70-hIFN-γ immunocytokine (thick solid line), followed by labeling with FITC-conjugated anti-human IgG secondary antibodies and detection of fluorescence by FACS. All four ATCC cell lines are robustly and specifically stained by both anti-CD70 IFN-γ immunocytokines.

Next, we determined if IFN-γ immunocytokines retained their ability to recognize CD70 on the surface of human cells. We overexpressed (in 293T cells) Myc-tagged human CD70 from a mammalian expression vector, and confirmed CD70 protein expression in lysates by immunoblotting cell extracts with an anti-Myc antibody (Fig. 4A, inset), and on the cell-surface by FACS staining with a FITC-conjugated anti-CD70 monoclonal antibody (Fig. 4A). When 293T cells expressing either Myc-CD70 or, as negative control, the empty vector (Vec) were incubated with IFN-γ immunocytokines and analyzed by FACS, both immunocytokines specifically labeled cells expressing Myc-CD70, but not cells transfected with the empty vector (Fig. 4B, right panels). An isotype control human IgG antibody (Rituximab, targeting the B cell antigen CD20) labeled both Vec- and CD70-expressing 293T cells equivalently (Fig. 4B, left), demonstrating that CD70 binding by IFN-γ immunocytokines was specific.

To extend these observations, we tested the capacity of IFN-γ-immunocytokines to bind CD70 on ATCC-derived human RCC cell lines. Each of the cell lines used in this study – ACHN, 786-O, 769-P and Caki-1– have been previously shown to express CD70 [33]. Consistent with our expectations, both IFN-γ immunocytokines – but not an isotype control antibody - labeled all four ATCC cell lines equivalently and robustly (Fig. 4C) These results demonstrate that IFN-γ immunocytokines are capable of binding CD70 on the surface of human RCC cells.

### Anti-CD70-IFN-γ Immunocytokines Exert Species-specific Cytotoxic Activity against RCC Cell Lines

Our ultimate reason for generating IFN-γ immunocytokines is to exploit IFN-γ’s direct tumoricidal effects for the treatment of RCC. Native IFN-γ induces RIP1 kinase-dependent necrotic cell death in cells rendered susceptible by genetic ablation of NF-κB [14]), or by treatment with sub-toxic doses of the proteasome inhibitor bortezomib (RJT, PC and SB, unpublished data). We therefore tested if each IFN-γ immunocytokine retained the IFN-γ-mediated ability to induce the death of RCC cells, when added to cultures of these cells in the presence of bortezomib.

**Figure 5:**
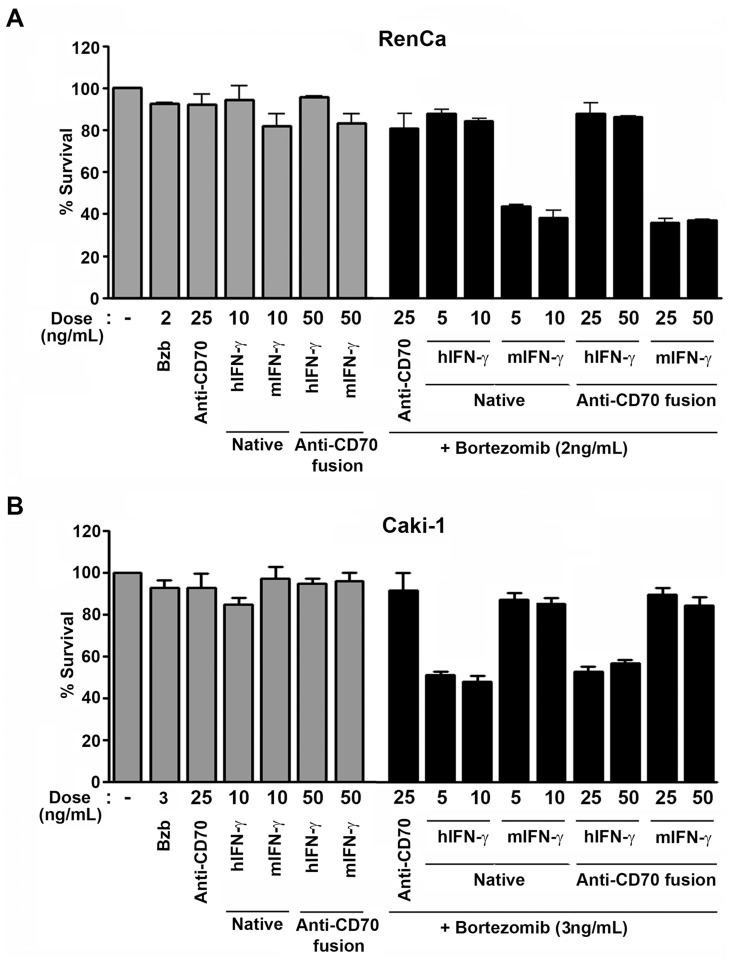
Anti-CD70-IFN-γ immunocytokines are cytotoxic to RCC cell lines in the presence of bortezomib. RCC cell lines RenCa (A) or Caki-1 (B) were treated either with unfused anti-CD70 antibody (‘Anti-CD70’), with recombinant, native human or murine IFN-γ (‘Native’), or with human or murine IFN-γ immunocytokines (‘Anti-CD70 fusion’) for 72 h in the presence of their MTD of bortezomib (black bars). As controls, these cells were also treated with each agent singly (grey bars). In conditions requiring bortezomib co-treatment, bortezomib was added to cells 1 h before IFN-γ.

For these experiments, we used RenCa (Fig. 5A) or Caki-1 (Fig. 5B) as representative examples of murine and human RCC cell lines, respectively, that respond functionally to IFN-γ (see Figs. 2 and 3). We first conducted a dose-finding study to determine the maximum tolerated dose (MTD) of bortezomib for each cell line, defined as the highest dose of this agent that was non-toxic to >85% of cells over a 72 h period. From this study, we identified doses of 2 ng/ml for RenCa and 3 ng/ml for Caki-1 as the MTD for bortezomib (Fig. 5).

Neither native murine nor human IFN-γ was significantly toxic to either cell line at doses of 10 ng/ml each over a 72 h period, when added to cells without bortezomib (Fig. 5, grey bars). Similarly, neither murine nor human IFN-γ immunocytokines exerted significant toxicity on either cell line over 72 h, when used by themselves at concentrations of 50 ng/ml (the molar equivalent of treating cells with 10 ng/ml native IFN-γ; Fig. 5, grey bars). By contrast, the same doses of both native IFN-γ and IFN-γ immunocytokines triggered species-specific cell death when added to RCC cells in the presence of the MTD of bortezomib (Fig. 5, black bars). The magnitude of cell death induced by IFN-γ immunocytokines was comparable to that induced by native IFN-γ in their respective target cells (∼70% cell death in RenCa and ∼50% in Caki-1), demonstrating that fusing IFN-γ to anti-CD70 antibody does not adversely affect its ability to induce death in susceptible cells. Notably, ∼100% of cells co-treated with bortezomib and IFN-γ immunocytokines succumbed to this combination when examined after a further 48 h, but interpretation of these data was confounded by toxicity arising from continued exposure to bortezomib alone (not shown).

### Anti-CD70-IFN-γ Immunocytokines Induce RIP1 Kinase-dependent Necrosis in RCC Cell Lines

**Figure 6:**
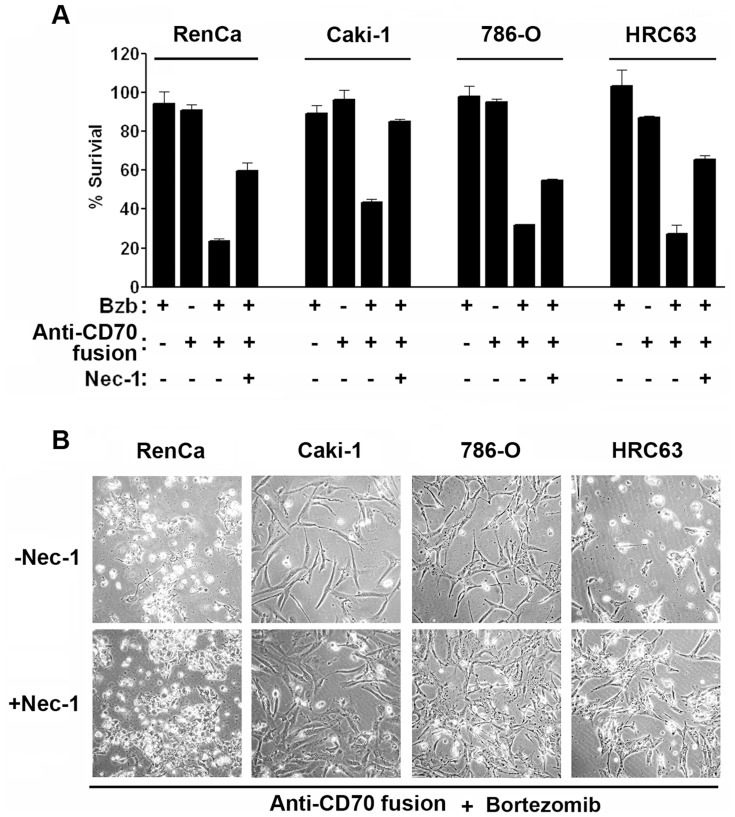
Anti-CD70-IFN-γ immunocytokines exert RIP1-dependent necrotic activity on RCC cell lines. (A) RenCa, Caki-1, 786-O, or HRC63 cells were co-treated with bortezomib (MTD) and, respectively, murine (RenCa) or human (Caki-1, 786-O, and HRC63) IFN-γ immunocytokines (‘Anti-CD70 fusion’, 50 ng/ml) in the presence or absence of 50 μM RIP1 kinase inhibitor Nec-1 for 72–84 h. The MTD of bortezomib for 786-0 and HRC63 cells was 4 ng/ml and 2 ng/ml, respectively. Cell viability was determined by Trypan Blue exclusion analysis. Error bars represent mean +/− S.D; n = 3. (B) RenCa, Caki-1, 786-O, or HRC63 cells pre-treated without (-Nec-1) or with (+Nec-1) for 1h, before co-treatment with IFN-γ immunocytokines and bortezomib as in (A), were photographed 72 h post-treatment.

We have previously shown that the dominant mechanism of cell death induced by native IFN-γ - in the setting of NF-κB inhibition - was RIP1 kinase-dependent necrosis [14]. To test if IFN-γ immunocytokines also induced RIP1-mediated necrotic death when combined with bortezomib, we pretreated (for 1h) RenCa and Caki-1 cells, as well as the additional pVHL-null human RCC cell lines 786-0 and HRC63, with a stable analog of the selective RIP1 kinase inhibitor Necrostatin-1 (Nec-1) [48], [49], before exposing these cells to the combination of IFN-γ immunocytokines and bortezomib. In all cases, Nec-1– but not an inactive analog (Nec-1i [49], not shown) provided significant protection against cell death induced by IFN-γ immunocytokines (Fig. 6 A,B). Altogether, these results show that IFN-γ immunocytokines can induce RIP1-kinase mediated necrosis in the RCC cell lines when survival signals are inhibited, and lend strong support to the idea that IFN-γ immunocytokines will allow the direct exploitation of IFN-γ’s tumoricidal potential in RCC.

### IFN-γ Signaling Components Show Elevated Expression in ccRCC

**Figure 7:**
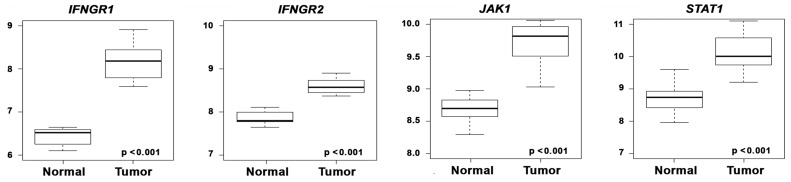
IFN-γ signaling components show elevated expression in ccRCC. Box plots depicting mRNA expression levels of key IFN-γ signaling components (*IFNGR1*, *IFNGR2*, *JAK1* and *STAT1*) in 21 paired ccRCC and normal samples [50]. The data were normalized using Robust Multi-array average (RMA) [54]. The gene fold-changes for Tumor-Normal comparison were obtained by LIMMA [41]. Y-axis shows RMA-normalized expression level (on log_2_ scale) of each mRNA. The solid line within each box is the median, and distance between box and whiskers indicate interquartile ranges.

Aside from bioavailability of IFN-γ fusions at the tumor, a key factor determining the balance between therapeutic efficacy as a tumoricidal agent versus side effects arising from non-specific activity of IFN-γ is distribution of the IFN-γ receptor. In other words, even if the anti-CD70 antibodies were to efficaciously target IFN-γ to RCC cells, the selective tumoricidal activity of this cytokine will still depend on intact IFN-γ signaling in RCC cells and, perhaps critically, on the ratio of IFN-γ receptor levels on RCC cells versus surrounding normal tissue. To this end, we evaluated the expression profiles of IFN-γ receptor subunits on primary patient-derived ccRCC samples, versus paired normal renal-tissue controls obtained from the same patient, in data obtained by Copland and colleagues and deposited in the Gene Expression Omnibus [50]. Examination of this rigorously-controlled dataset revealed that both IFN-γ receptor subunits, as well as the key downstream signaling intermediates (Jak1 and STAT1) displayed significantly greater expression in ccRCC samples, compared to controls (Fig. 7). Although the reason(s) for elevated expression of IFN-γ signaling components is currently unclear, these data allow us to predict that IFN-γ’s effects will remain largely specific to RCC cells once delivered to the tumor.

## Discussion

In this report, we describe the development and characterization of two antibody-IFN-γ fusion chimeras, or ‘immunocytokines’, both targeting the RCC biomarker CD70, but one bearing the human cytokine and the other, the murine ortholog of IFN-γ. The immunocytokines demonstrated CD70 binding and IFN-γ activity comparable to, respectively, unfused anti-CD70 antibody and recombinant human and murine IFN-γ. Importantly, both IFN-γ immunocytokines demonstrated *in vitro* IFN-γ-dependent cytotoxic activity on RCC cells, when combined with the proteasome inhibitor bortezomib. These observations provide evidence that anti-CD70-IFN-γ immunocytokines will enable the unmasking of IFN-γ’s direct cytotoxic properties for the treatment of metastatic RCC.

Although IFN-γ shares many similar biological properties with type I (α/β) IFNs (e.g. the long-time RCC biotherapeutic IFN-α), its use as an anti-RCC agent in the clinic has been underwhelming. In particular, a randomized phase III trial employing IFN-γ as a monotherapy for metastatic RCC failed to show any benefit over placebo [51]. While these results are disappointing, we wish to draw attention to two issues limiting the efficacy of IFN-γ as anti-RCC biotherapeutic in this and other trials. First, the underlying rationale of the phase III trial, as well as of most other trials employing IFN-γ in RCC, was sole exploitation of IFN-γ’s immunomodulatory properties; consequently, this cytokine’s direct anti-tumor (e.g. anti-proliferative and cytotoxic) properties remain mostly untapped. Second, previous phase I trials have demonstrated that single-dose intravenous bolus therapy is handicapped by the poor pharmacokinetics (half-life in circulation of ∼25–35 minutes) of recombinant cytokine, with consequent dose-limiting toxicity arising from unnecessarily high doses [24], [25].

Epstein and colleagues had previously shown that both these shortcomings can be overcome by fusing IFN-γ to tumor-targeted antibodies [28], [29]. These fusions were shown to not only stabilize IFN-γ in circulation, but to also deliver IFN-γ to the tumor at bioactive concentrations [28], [29]. Here, we demonstrate that IFN-γ, when fused to anti-CD70 antibody, effectively labels and kills CD70-bearing RCC cells *in vitro*. In future experiments, we will test if these fusions are similarly effective in targeting RCC cells *in vivo*, where IFN-γ’s direct anti-tumor effects – and its pro-necrotic ability in particular - can be exploited without significant toxicity issues arising from systemic distribution of IFN-γ. It is noteworthy that, compared to normal surrounding tissue, RCC cells display elevated levels of IFN signaling components, including of the IFN-γ receptor IFNGR. Although the underlying reasons for such an RCC-selective increase in IFNGR expression is currently unclear, these unexpected findings further support to the notion that the effects of IFN-γ fusions will be tumor-selective.

The strategy of targeting cytokines to tumor cells in order to exploit their tumoricidal effects is not limited to IFN-γ. Data from Pfeffer and colleagues shows that NF-κB is a survival factor against type I (α/β) IFNs as well, and neutralizing NF-κB in various tumor cells sensitizes them to apoptotic death by IFN-α [52]. Further, Morrison and colleagues have reported that fusing IFN-α to rituximab directly induces apoptosis in multiple myeloma cells *in vivo* [53]. Extending these findings, we have observed that both IFN-α and IFN-β can induce necrotic death in dividing cells when survival signals are disabled (not shown). Thus, both type I and type II IFNs – in addition to modulating the anti-tumor immune response - can engage multiple cytotoxic mechanisms if directly delivered to tumors. Cumulatively, these results raise the exciting possibility that the tumoricidal properties of IFNs can be exploited by combining agents that selectively neutralize tumor-cell survival signaling with IFN fusions that target any of the >20 highly-aggressive cancers for IFNs are currently approved by the United States Food and Drug Administration.
